# Bell’s palsy and influenza(H1N1)pdm09 containing vaccines: A self-controlled case series

**DOI:** 10.1371/journal.pone.0175539

**Published:** 2017-05-03

**Authors:** Leonoor Wijnans, Caitlin N. Dodd, Daniel Weibel, Miriam Sturkenboom

**Affiliations:** 1 Department of Medical Informatics, Erasmus University Medical Centre, Rotterdam, The Netherlands; 2 Medicines Evaluation Board, Utrecht, The Netherlands; University of Waterloo, CANADA

## Abstract

**Background:**

An association between AS03 adjuvanted pandemic influenza vaccine and the occurrence of Bell’s palsy was found in a population based cohort study in Stockholm, Sweden. To evaluate this association in a different population, we conducted a self-controlled case series in a primary health care database, THIN, in the United Kingdom. The aim of this study was to determine whether there was an increased risk of Bell’s palsy following vaccination with any influenza vaccine containing A/California/7/2009 (H1N1)-like viral strains. Secondly, we investigated whether risks were different following pandemic influenza A(H1N1)pdm09 vaccines and seasonal influenza vaccines containing the influenza A(H1N1)pdm09 strain.

**Methods:**

The study population comprised all incident Bell’s palsy cases between 1 June 2009 and 30 June 2013 identified in THIN. We determined the relative incidence (RI) of Bell’s palsy during the 6 weeks following vaccination with either pandemic or seasonal influenza vaccine. All analyses were adjusted for seasonality and confounding variables.

**Results:**

We found an incidence rate of Bell’s palsy of 38.7 per 100,000 person years. Both acute respiratory infection (ARI) consultations and pregnancy were found to be confounders. When adjusted for seasonality, ARI consultations and pregnancies, the RI during the 42 days after vaccination with an influenza vaccine was 0.85 (95% CI: 0.72–1.01). The RI was similar during the 42 days following seasonal vaccine (0.96, 95%CI: 0.82–1.13) or pandemic vaccine (0.73, 95%CI: 0.47–1.12).

**Conclusion:**

We found no evidence for an increased incidence of Bell’s palsy following seasonal influenza vaccination overall, nor for monovalent pandemic influenza vaccine in 2009.

## Introduction

Bell’s palsy is an idiopathic peripheral-nerve palsy affecting the cranial nerve and the most common cause for facial paralysis [1] with an incidence between 15 to 50 cases per 100,000 people per year [1–3]. It is characterized by acute onset, unilateral facial paralysis, numbness or pain around the ear, reduction in taste and hypersensitivity to sounds. The diagnosis is made after excluding other possible causes for facial paralysis, including congenital, genetic and acquired causes. Standard diagnostic criteria are not available [4]. Bell’s palsy resolves spontaneously without treatment in most patients within 6 months. Some patients experience long-term sequelae with incomplete return of facial motor function and synkinesis [1]. The cause of Bell’s palsy is unknown. Inflammation is thought to play an important role in the aetiology of Bell’s palsy [1] and an auto-immune aetiology has also been suggested [5]. Known risk factors for Bell’s palsy include diabetes, a weakened immune system and pregnancy [1, 6].

Bell’s palsy has been associated with influenza vaccines in the past [7–12]. A large population-based study in the UK did not detect a relationship between inactivated influenza vaccines and Bell’s palsy [13], nor did a recent study in the US in children [14]. Due to the earlier associations and the unknown aetiology, Bell’s palsy remains an adverse event of interest following influenza vaccination.

Following the 2009/2010 influenza A(H1N1) pandemic, an association with Bell’s palsy was found with an AS03 adjuvanted pandemic influenza vaccine, Pandemrix, in a population based cohort study in Stockholm, Sweden with a hazard ratio (HR) of 1.25, 95% CI 1.06 to 1.48 [15]. The risk was highest during the first 6 weeks following vaccination (HR: 1.60, 1.25 to 2.05) and particularly present in those vaccinated early in the campaign (HR: 1.74, 95% CI 1.16 to 2.59), which were those with more (severe) underlying co-morbidity. Similarly, a signal was detected for monovalent pandemic influenza vaccines used in the Vaccine Safety Datalink (VSD) Project in the US in adults over the age of 25 years with a relative risk of 1.6 [16]. This last signal was not verified in a case centred analysis which found and odds ratio of 1.21 (95% CI: 0.93–1.57). Finally, a signal of an increased risk of Bell’s palsy during the 42 days after vaccination with pandemic (H1N1) 2009 vaccine was detected in Taiwan [17].

In order to evaluate the potential association of Bell’s palsy following influenza A(H1N1)pdm09 vaccination in a different population, we conducted a self-controlled case series study. The aim of this study was to determine whether there was an increased risk of Bell’s palsy following vaccination with any influenza vaccine containing A/California/7/2009 (H1N1)-like viral strains. Secondly, we looked whether risks were different following pandemic influenza A(H1N1)pdm09 vaccines and seasonal influenza vaccines containing the influenza A(H1N1)pdm09 strain.

## Methods

We used a self-controlled case series (SCCS) [18, 19] design in The Health Improvement Network (THIN) database. THIN includes data from 562 general practices across the UK and the population covered by THIN is representative of the UK population. The data in THIN have been validated for pharmacoepidemiology studies [20, 21].

### Study population, study period and outcome

The study population comprised all incident Bell’s palsy cases between 1 June 2009 and 30 June 2013 identified in THIN, from a total population in this time period of nearly 6 million. A Bell’s palsy case was defined as a person who had a consultation with a READ diagnosis code for Bell’s palsy (F310.00). Multiple cases per person were allowed. If diagnosis dates were more than 6 months apart, they constituted two separate cases. Considering the relatively high predictive value of over 75% of READ diagnosis codes for Bell’s palsy [13] no validation on identified cases was performed.

### Exposures

Influenza vaccination was identified through relevant codes and recorded by year and vaccine type (seasonal or pandemic), including pandemic influenza vaccination and seasonal influenza vaccinations for the years 2009/2010, 2010/2011, 2011/2012 and 2012/2013. In the UK both Pandemrix and Celvapan were used during the 2009–2010 influenza A(H1N1) pandemic, and information on brand was retrieved if available. Moreover, during the 2009–2010 season, persons could have received both a seasonal vaccine and a pandemic influenza vaccine. In theory these could have been given on the same day or close together making it difficult to attribute the risk to either. Considering the study by Stowe *et al* [13] no increased risk was expected for the seasonal vaccine, therefore this was disregarded in the primary analysis.

Because each person serves as his or her own control, stable confounders such as gender, genetics, socio-economic status, and underlying disease are controlled for. Covariates that were considered as potential confounders were calendar time, occurrence of acute respiratory infections (ARI), influenza diagnoses, and pregnancy. Considering the short observation period, no age effect was expected. ARI episodes and influenza diagnoses were identified by relevant READ codes (provided in S1 Table). Consultations for ARI or influenza occurring within 28 days of a previous consultation were excluded as likely related to the same episode. The risk window for ARI and influenza was 0 to 7 days following the date of infection. Pregnancies were identified by the date of delivery. The risk period was the 270 days (9 months) before the date of delivery.

### Analysis

We used means and standard deviations to describe continuous variables. For categorical variables, we used counts and percentages. We calculated the incidence rate of codes for Bell’s palsy using all person time in the database within the study period and similarly determined vaccination rates per season using all subjects in the database.

All descriptive statistics were compared between vaccinated cases and unvaccinated cases using t-tests for continuous variables and chi-squared tests for categorical variables. Associations between pregnancy, ARI consultations and influenza diagnoses and Bell’s palsy and influenza vaccination were determined. We determined the relative incidence (RI) of Bell’s palsy during the 6 weeks following vaccination with either pandemic or seasonal influenza vaccine using a conditional Poisson regression conducted on Bell’s palsy cases only. The risk period of interest was from days one to 42 following vaccination (D1 to D42), as this was the period with the highest risk found by Bardage et al [15]. As vaccination could be delayed following an episode of Bell’s palsy, the 14 days prior to vaccination were treated as a separate risk period in the analysis. The day of vaccination (D0) was also regarded as a separate risk period. Relative incidence of Bell’s palsy associated with pregnancy, influenza diagnosis, and ARI were estimated using the SCCS method [18]. If these univariate associations were significant, pregnancy, influenza, and ARI would be included as additional exposures in the primary analysis. All analyses were adjusted for calendar time by quarter.

Relative incidences were calculated separately for pandemic and for seasonal influenza vaccines, and for each season (vaccination period). All person time was included in analysis of risk following any pH1N1-containing vaccine exposure while person time was limited to October 1 to 61 days following the last administration in separate analyses of pandemic H1N1 vaccine and seasonal vaccines. As less than 0.1% of vaccinated cases received Celvapan during the 2009/2010 pandemic [22], we consider the findings with pandemic vaccines in our study to be applicable to Pandemrix. Age and sex specific relative incidences of Bell’s palsy within 6 weeks of influenza vaccination were calculated.

To further account for the risk of deferral of vaccination after receiving a diagnosis of Bell’s palsy, we performed sensitivity analyses in which only the observation time after vaccination was considered.

All analyses were conducted using SAS 9.2.

## Results

We identified 6381 Bell’s palsy cases in 6288 persons. Of these, 6198 persons had one code for Bell’s palsy, 87 persons had two and three persons had three consultations with a Bell’s palsy code during the study period. The incidence rate was 38.7 per 100,000 person years (Table 1)

**Table 1:** Main characteristics of THIN population overall and by case status.

	Non-Case n = 5,726,368		Case n = 6,288		Total n = 5,732,656	
*Demographics*						
Female (n (%))	2,913,751	(50.88)	3,194	(50.80)	2,917,343	(50.88)
Mean Follow Up Time in years (SD)	3.13	(1.58)	3.72	(1.27)	3.13	(1.58)
Mean age in years (SD)	37.10	(23.32)	45.00	(20.20)	37.11	(23.32)
Age (n (%))						
<45 yrs	3,646,538	(63.68)	3,217	(51.16)	3,650,127	(63.67)
45–65 yrs	1,289,596	(22.52)	1,949	(31.00)	1,291,825	(22.53)
>65 yrs	790,234	(13.80)	1,122	(17.84)	791,495	(13.80)
≥1 ARI episode (n (%))	1,489,391	(26.01)	2231	(35.48)	1,491,622	(26.02)
≥ 1Pregnancy (n (%))	122,878	(2.15)	158	(2.51)	123,036	(2.15)
2009 Pandemic (n(%))	524059	(9.15)	901	(14.33)	524960	(9.16)
Seasonal 2009–2010 (n(%))	913,250	(15.95)	1660	(26.40)	914,910	(15.96)
Seasonal 2010–2011 (n(%))	789912	(13.79)	1529	(24.32)	791,411	(13.80)
Seasonal 2011–2012 (n(%))	856684	(14.93)	1747	(27.78)	856,684	(14.94)
Seasonal 2012–2013 (n(%))	817751	(14.28)	1702	(27.07)	819,453	(14.29)

**Fig 1:**
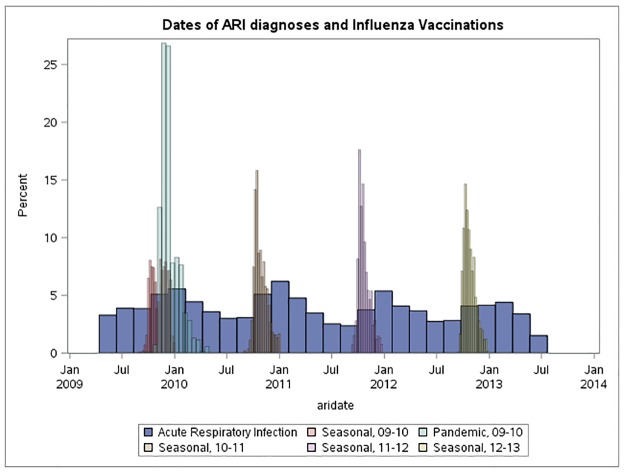
Distribution of Bell’s palsy diagnosis dates and dates of vaccination during the observation period.

The characteristics of the cases by vaccination status (seasonal and pandemic) are presented in Table 2. Cases who were vaccinated with either pandemic or seasonal influenza vaccine were older and had more consultations for ARI during the study period. Moreover, cases who received seasonal influenza vaccines were more likely to be female. The distribution of Bell’s palsy dates relative to vaccination dates is presented in Fig 1.

**Table 2:** Main characteristics of cases occurring between 1 June 2009 and 30 June 2013 by vaccination status.

	Received seasonal vaccine*					Received pandemic vaccine				
	Yes n = 2408		No n = 3880		*p-value*	Yes n = 901		No n = 5387		*p-value*
*Demographics*										
Female (n (%))	1313	(54.53)	1881	(48.48)	< .0001	454	(50.39)	2740	(50.86)	0.79
Mean age in years (SD)	58.59	(18.07)	36.56	(16.51)	< .0001	56.75	(19.64)	43.03	(19.61)	< .0001
Age (n (%))										
<45 yrs	532	(22.09)	2685	(69.20)		212	(23.53)	3005	(55.78)	
45–65 yrs	897	(37.25)	1052	(27.11)		357	(39.62)	1592	(29.55)	
>65 yrs	979	(40.66)	143	(3.69)	< .0001	332	(36.85)	790	(14.66)	< .0001

Of cases, 14% received the monovalent pandemic influenza vaccine whilst seasonal vaccines were received by 24 to 28% of cases dependent on the year. Thirty-five percent (2232 persons) experienced at least one episode of ARI during follow-up. In total, 3.5% (220 cases) received an influenza diagnosis. During follow-up 155 women had one pregnancy (4.85%) and three women had two pregnancies (0.09%).

**Fig 2:**
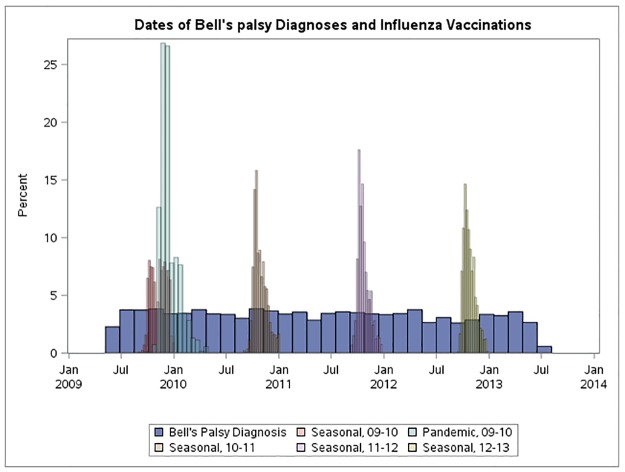
Distribution of acute respiratory infection dates and dates of vaccination during the observation period. There was no statistical evidence of an association between Bell’s palsy and a consultation for influenza (RR 2.41, 95% CI 0.76–7.58).

We found that pregnancy was associated with a higher risk of Bell’s palsy (RR 1.75, 95% CI 1.19–2.57) and that pregnant women had a higher likelihood of receiving an influenza vaccine (RR 5.05, 95% CI 3.26–7.82). An episode of ARI was strongly associated with Bell’s palsy on the day of consultation (RR 6.99, 95% CI: 4.39–11.13), but also in the 7 days following a consultation for ARI (RR 2.44, 95% CI 1.81–3.30). In addition, an episode of ARI was associated with an increased incidence of vaccination on the day of consultation for ARI (RR 2.93, 95% CI 1.58–5.46) and a reduced incidence of vaccination during the week following a consultation for ARI (RR 0.50, 95% CI 0.28–0.89). The distribution of ARI dates relative to vaccination dates over calendar time is given in Fig 2.

The crude RI of Bell’s palsy during the 42 days after vaccination with an influenza vaccine was 0.88 (95% CI: 0.74–1.04). On the day of vaccination the relative incidence was 2.15 (95% CI: 1.12–4.14). The RI was reduced in the fourteen days prior to vaccination, 0.70 (95% CI: 0.51–0.96). When adjusted for seasonality, episodes of ARI and pregnancies, the RI during the 42 days after vaccination with an influenza vaccine was 0.85 (95% CI: 0.72–1.01). At the date of vaccination the adjusted RI was 2.08 (95% CI: 1.08–4.01), during the 14 days preceding vaccination the adjusted RI was 0.68 (95% CI: 0.50–0.93).

When considering the type of vaccine (i.e. seasonal vs. pandemic) the adjusted RI was similar during the 42 days following seasonal vaccine (0.96, 95%CI: 0.82–1.13) or pandemic vaccine (0.73, 95%CI: 0.47–1.12).

The adjusted (for ARI and seasonality in men; for ARI, seasonality, and pregnancy in women) RI during the 42 days following influenza vaccination (any) was slightly lower in women (0.77, 95% CI: 0.61–0.99) compared to men (0.94, 95% CI: 0.74–1.19), with confidence intervals overlapping.

The adjusted relative incidence of Bell’s palsy within 42 days of influenza vaccine stratified by vaccine and age can be found in Table 3.

**Table 3:** Age and season specific relative incidences (95% CI) of Bell’s palsy within 42 days of influenza vaccination (adjusted for seasonality, ARI consultations and pregnancy).

Risk Period	All age groups				Age <45			Age 45 to 64			Age 65 +		
	Person Time (yrs)	N	RI	95% CI	N	RI	95% CI	N	RR	95% CI	N	RR	95% CI
Any vaccine*													
Day -14 to -1	244	41	0.68	0.50–0.93	7	0.92	0.43–1.95	15	0.73	0.44–1.23	19	0.58	0.36–0.92
Day 0	17	9	2.08	1.08–4.01	2	3.58	0.89–14.40	2	1.36	0.34–5.44	5	2.12	0.88–5.15
Day 1 to 42	733	154	0.85	0.72–1.01	25	1.07	0.71–1.62	45	0.73	0.53–1.00	84	0.86	0.67–1.10
Non-Risk	23,031	5629											
2009 Pandemic													
Day -14 to -1	39	2	0.18	0.05–0.74	1	0.41	0.06–2.98	1	0.23	0.03–1.65	0	NA	NA
Day 0	3.2	0	NA	NA	0	NA	NA	0	NA	NA	0	NA	NA
Day 1 to 42	118	24	0.73	0.47–1.12	3	0.40	0.12–1.28	9	0.70	0.35–1.43	12	0.93	0.50–1.74
Non-Risk	5076	3681											
Season 2010–2011													
Day -14 to -1	52	7	0.64	0.29–1.39	0	NA	NA	1	0.36	0.05–2.68	6	0.90	0.37–2.17
Day 0	4.4	2	2.25	0.55–9.18	0	NA	NA	1	4.36	0.58–32.57	1	1.86	0.25–13.64
Day 1 to 42	198	53	1.28	0.90–1.80	11	1.58	0.73–3.42	16	1.44	0.75–2.76	26	1.03	0.62–1.72
Non-Risk	2705	635											
Season 2011–2012													
Day -14 to -1	54	17	1.23	0.73–2.08	3	1.28	0.36–4.53	7	2.17	0.91–5.16	7	0.76	0.34–1.72
Day 0	4.9	1	0.83	0.12–5.98	1	5.39	0.71–41.15	0	NA	NA	0	NA	NA
Day 1 to 42	224	45	0.81	0.57–1.16	5	0.59	0.21–1.60	14	1.12	0.58–2.19	26	0.67	0.41–1.10
Non-Risk	2522	537											
Season 2012–2013													
Day -14 to -1	54	8	0.62	0.30–1.30	3	1.82	0.51–6.50	2	0.34	0.08–1.44	3	0.58	0.17–1.93
Day 0	4.8	3	2.87	0.91–9.09	1	6.86	0.89–52.67	1	2.22	0.30–16.25	1	2.33	0.32–17.23
Day 1 to 42	217	44	0.90	0.63–1.28	9	1.33	0.58–3.07	9	0.43	0.21–0.88	26	1.28	0.74–2.20
Non-Risk	2627	547											

All Bell’s palsy cases regardless of vaccination status included. All analyses adjusted for seasonality by quarter, ARI consultations, and pregnancy in strata that contained pregnant cases.

In the analysis in which only observation time after vaccination was included, exposure to 2009 pH1N1 vaccine with control for ARI, seasonality, and pregnancy produced a RI of 0.88 (95% CI: 0.47, 1.65) while exposure to 2010–11, 2011–12, and 2012–13 seasonal vaccines produced RIs of 1.56 (0.95, 2.57); 0.69 (0.45, 1.06); and 0.91 (0.57, 1.46), respectively.

The main analysis disregarded seasonal influenza vaccines during the 2009–2010 season. We considered the receipt of seasonal influenza vaccines during the 2009–2010 season as a separate risk factor in a sensitivity analysis. The results showed a RI of 1.14 (0.86, 1.51) in the 42 days following vaccination.

## Discussion

Bell’s palsy is a syndrome for which the exact cause is unclear. As a result it could have multiple triggers, of which—considering the hypothetical autoimmune aetiology—influenza and influenza vaccination could be one. Clusters of Bell’s palsy cases have been reported following influenza vaccination in the past. An association was reported for Bell’s palsy and Pandemrix, an AS03 adjuvanted pandemic influenza vaccine in Sweden [15], and a signal was reported from Taiwan [17]. In this study, we evaluated the risk of Bell’s palsy following vaccination with influenza vaccines containing A/California/7/2009 (H1N1)-like viral strains, including pandemic vaccines, in the UK.

The increased risk of Bell’s palsy on the day of influenza vaccination was expected, based upon the findings of Stowe et al [13], and a likely opportunistic recording of cases.

We found no evidence of an increased incidence of Bell’s palsy consultations following seasonal influenza vaccination overall, nor for monovalent pandemic influenza vaccine in 2009. Therefore our study does not confirm the results identified by Bardage et al [15] in Sweden. While Bardage et al. controlled for sex, age, and health utilization as measured by contacts within the year prior to vaccination, they were unable to control for unmeasured within-person confounders or for seasonality. Given the association we found here between ARI and Bell’s palsy, failure to control for infection and/or seasonality could lead to the increased HR found by Bardage et al. When adjusted for seasonality, episodes of ARI and pregnancies, the RI during the 42 days after vaccination with an influenza vaccine was 0.85 (95% CI: 0.72–1.01).

Other than mild protective effects in women following exposure to any H1N1-containing vaccine and in 45–64 year olds following exposure to the 2012–13 seasonal vaccine, all estimated RIs associated with vaccine exposure were non-significant. Given the number of associations assessed together with the upper limits of the confidence intervals being nearly equal to one, we did not further investigate these apparent protective effects, as these could reasonably be due to chance.

One of the more restrictive assumptions of the SCCS method is that the distribution of exposure after a certain time must be independent of the event history prior to that time [18]. Bell’s palsy is not a contra-indication for influenza vaccination. Nonetheless, it is possible that people will delay vaccination after Bell’s palsy, which can represent a violation of the assumption of the SCCS. Generally, this delay in vaccination would bias RI estimates upward by producing a scarcity of cases in control intervals. In our main analysis we fixed the 14 days prior to vaccination as a separate risk period. The reduced incidence found during this risk period demonstrates that persons will delay vaccination when diagnosed with Bell’s palsy. We assumed that a 14 day period would be sufficient to exclude any bias resulting from this delay. As evidenced by the sensitivity analysis which only considered observation time after vaccination and produced estimates very similar to those produced with a 14-day low risk period, this 14-day period was sufficient to control for a potential healthy vaccinee effect.

A second restrictive assumption of the SCCS method is that events are either recurrent and independent within individuals or not-recurrent and uncommon [18]. Bell’s palsy can recur, however this is rare [1] and is reported to do after a latency period of approximately 10 years [5, 13]. In our study we considered any second consultation of Bell’s palsy within 6 months to belong to a single episode. We found that 1.4% of persons had more than one episode within our relatively short observation period. As recurrent events are rare we assume the bias is negligible [23].

Our study has limitations that could impact the observed results.

Whilst the SCCS inherently deals with measured and unmeasured fixed confounding variables, time varying confounders will still need to be measured and adjusted for. We adjusted for seasonality by quarter, consultations for ARI and pregnancies, as these factors were identified as confounders in our study. Although both ARI and pregnancies were associated with exposure and outcome, adjusting for them had minimal impact on estimates. As we identified pregnancies by date of delivery we did not capture all pregnancies. Similarly, consultations for ARI do not reflect all the ARIs actually occurring. We could not adjust for time varying factors that were not measured such as changes in medical coding practice, healthcare seeking behaviour, or vaccination policy.

Finally, persons who develop Bell’s palsy may consult their GP only after prolonged persistence of symptoms or not at all, making incomplete reporting of cases possible in our study. If reporting was differential by vaccination status, meaning if persons who develop Bell’s palsy shortly following vaccination were more likely or less likely to consult their GP, this would have introduced bias in this study.

In conclusion, our study did not provide evidence of an increased risk of Bell’s palsy following vaccination with any influenza vaccine containing A/California/7/2009 (H1N1)-like viral strains, either pandemic or seasonal vaccines.

## Supporting information

S1 Table Read codes for data extraction. (DOCX)
