# Supplementary material for: Bell’s palsy and influenza(H1N1)pdm09 containing vaccines: A self-controlled case series
Source: PLoS One. 2017 May 3;12(5):e0175539. doi: 10.1371/journal.pone.0175539 (PMC5414992; doi:10.1371/journal.pone.0175539)
Supplement: S1 Table — (DOCX) [file pone.0175539.s001.docx]

# Supporting Information 1: READ CODES FOR DATA EXTRACTION

**Read code for Bell’s palsy**

| F31..00 | Facial nerve disorders |
| --- | --- |
| **F310.00** | **Bell's (facial) palsy** |

**AHD codes for influenza vaccination**

| 1002090105 | Influenza A H1N1v |
| --- | --- |
| 1002090104 | Influenza A H1N1v unknown brand (other health provider) |
| 1002090103 | Influenza A H1N1v (other health provider) |
| 1002090102 | Influenza A H1N1v (other health provider) |
| 1002090101 | Influenza A H1N1v |
| 1002090100 | Influenza A H1N1v unknown brand |
| 1002090000 | Influenza |

**READ codes for influenza vaccination**

| 65E..00 | Influenza vaccination |
| --- | --- |
| 65E0.00 | First pandemic influenza vaccination |
| 65E1.00 | Second pandemic influenza vaccination |
| 65E2.00 | Influenza vacc othr hlth prov |
| 65E3.00 | 1st pan flu vac othr hlth prov |
| 65E4.00 | 2nd pan flu vac othr hlth prov |
| 65E5.00 | CELVAPAN - first influenza A (H1N1v) 2009 vaccination given |
| 65E6.00 | CELVAPAN - second influenza A (H1N1v) 2009 vaccination given |
| 65E7.00 | CELVAPAN - 1st flu A (H1N1v) 2009 vacc by othr hlth provider |
| 65E8.00 | CELVAPAN - 2nd flu A (H1N1v) 2009 vacc by othr hlth provider |
| 65E9.00 | PANDEMRIX - first influenza A (H1N1v) 2009 vaccination given |
| 65EA.00 | PANDEMRIX - second influenza A (H1N1v) 2009 vaccination give |
| 65EB.00 | PANDEMRIX - 1st flu A (H1N1v) 2009 vac by othr hlth provider |
| 65EC.00 | PANDEMRIX - 2nd flu A (H1N1v) 2009 vac by othr hlth provider |

**READ codes for ARI**

| 'H00..00' | 'H03..00' | 'H042000' | 'H060700' | 'H01yz00' | 'H040000' | 'H053.00' | 'H061200' |
| --- | --- | --- | --- | --- | --- | --- | --- |
| 'H00..11' | 'H03..11' | 'H042100' | 'H060800' | 'H01z.00' | 'H040100' | 'H055.00' | 'H061300' |
| 'H00..12' | 'H03..12' | 'H042z00' | 'H060900' | 'H02..00' | 'H040200' | 'H05y.00' | 'H061400' |
| 'H00..13' | 'H030.00' | 'H043.00' | 'H060A00' | 'H02..11' | 'H040300' | 'H05z.00' | 'H061500' |
| 'H00..15' | 'H031.00' | 'H043.11' | 'H060B00' | 'H02..12' | 'H040400' | 'H05z.11' | 'H061600' |
| 'H00..16' | 'H032.00' | 'H043000' | 'H060C00' | 'H02..13' | 'H040500' | 'H05z.12' | 'H061z00' |
| 'H01..00' | 'H033.00' | 'H043100' | 'H060D00' | 'H020.00' | 'H040600' | 'H06..00' | 'H062.00' |
| 'H01..11' | 'H034.00' | 'H043200' | 'H060E00' | 'H021.00' | 'H040w00' | 'H060.00' | 'H06z.00' |
| 'H010.00' | 'H035.00' | 'H043211' | 'H060F00' | 'H022.00' | 'H040x00' | 'H060.11' | 'H06z000' |
| 'H010.11' | 'H035000' | 'H043z00' | 'H060v00' | 'H023.00' | 'H040z00' | 'H060000' | 'H06z011' |
| 'H011.00' | 'H035100' | 'H044.00' | 'H060w00' | 'H023000' | 'H041.00' | 'H060100' | 'H06z100' |
| 'H012.00' | 'H035z00' | 'H04z.00' | 'H060x00' | 'H023100' | 'H041000' | 'H060200' | 'H06z111' |
| 'H013.00' | 'H036.00' | 'H05..00' | 'H060z00' | 'H023z00' | 'H041100' | 'H060300' | 'H06z112' |
| 'H014.00' | 'H03z.00' | 'H050.00' | 'H061.00' | 'H024.00' | 'H041z00' | 'H060400' | 'H07..00' |
| 'H01y.00' | 'H04..00' | 'H051.00' | 'H061000' | 'H025.00' | 'H042.00' | 'H060500' | 'H0y..00' |
| 'H01y000' | 'H040.00' | 'H052.00' | 'H061100' | 'H02z.00' | 'H042.11' | 'H060600' | 'H0z..00' |

**READ codes for delivery date**

| '63...00' | '63...00' | '639..00' | '63A..00' | '632..00' | '63D..00' |
| --- | --- | --- | --- | --- | --- |
| '6331.00' | '633..00' | '633a.00' | '6341.00' | '6342.00' | '63E2.00' |
| '635..11' | '7F10.00' | '7F10000' | '7F10100' | '7F10y00' | '7F10z00' |
| '7F10z11' | '7F10z12' | '7F11.00' | '7F11000' | '7F11100' | '7F11200' |
| '7F11300' | '7F11y00' | '7F11z00' | '7F12.00' | '7F12000' | '7F12100' |
| '7F12111' | '7F12y00' | '7F12z00' | '7F13.00' | '7F13000' | 7F13100 ' |
| '7F13111' | '7F13200' | '7F13300' | '7F13y00' | '7F13z00' | 7F14.00' |
| '7F14100' | '7F14y00' | '7F14z00' | '7F15.00' | '7F15000' | '7F14000' |
| '7F15100' | '7F15y00' | '7F15z00' | '7F16.00' | '7F16000' | '7F16200' |
| '7F16300' | '7F16400' | '7F16500' | '7F16600' | '7F16700' | '7F16800' |
| '7F16900' | '7F16A00' | '7F16B00' | '7F16y00' | '7F16z00' | '7F17.00' |
| '7F17000' | '7F17100' | '7F17200' | '7F17300' | '7F17y00' | '7F17z00' |
| '7F18.00' | '7F18000' | '7F18100' | '7F16100' | '7F18y00' | '7F18z00' |
| '7F19.00' | '7F19000' | '7F19100' | '7F19y00' | '7F19z00' | '7F1A.00' |
| '7F17.11' | '7F17.12' | 'L34..00' | 'L398.00' | 'L398300' | 'L398400' |
| 'Ly0..00' |  |  |  |  |  |

**READ codes for influenza**

| H27.. | H27z. | H270z | H270. |
| --- | --- | --- | --- |
| Hyu06 | H27yz | H27y. | H2710 |
| H2711 | H27yz | H27y. | H2710 |
| H2711 | Hyu07 | H27y1 | Hyu05 |
| H271. | H27y0 | H271z |  |
